# First report of *Sarcocystis halieti* (Apicomplexa) in bearded vulture (*Gypaetus barbatus*)

**DOI:** 10.1007/s11259-023-10191-1

**Published:** 2023-08-09

**Authors:** Petras Prakas, Josep Estruch, Roser Velarde, Mikas Ilgūnas, Donatas Šneideris, Olga Nicolás-Francisco, Ignasi Marco, Rafael Calero-Bernal

**Affiliations:** 1 Nature Research Centre, Akademijos 2, Vilnius, 08412 Lithuania; 2 Wildlife Ecology and Health group (WE&H) and Servei d’Ecopatologia de Fauna Salvatge (SEFaS), Departament de Medicina i Cirurgia Animals, Facultat de Veterinària, Universitat Autònoma de Barcelona, Bellaterra, Barcelona, 08193 Spain; 3 Forestal Catalana, Ministry of Climate Action, Food and Rural Agenda (Government of Catalonia), Lleida, 25595 Spain; 4 Wildlife Conservation Medicine group (WildCoM), Departament de Medicina i Cirurgia Animals, Facultat de Veterinària, Universitat Autònoma de Barcelona, Bellaterra, Barcelona, 08193 Spain; 5 Department of Animal Health, Faculty of Veterinary, SALUVET, Complutense University of Madrid, Madrid, 28040 Spain

**Keywords:** Apicomplexa, *Sarcocystis*, Raptorial birds, ITS1, Phylogeny

## Abstract

At least three *Sarcocystis* species (*S. falcatula, S. halieti* and *S. wobeseri*–like) have been detected infecting raptorial birds. By histopathology and PCR-sequencing of the ITS1 marker, *S. halieti* was detected in a bearded vulture (*Gypaetus barbatus*) and a black kite (*Milvus migrans*) from the Catalonia region in North Spain. The 241 bp-long sequences obtained from the *Sarcocystis* organisms detected in both raptors showed 97.5–99.6% and 97.9–100% similarity with those of previously identified *S. halieti*; also, the phylogenetic trees generated placed the identified sequences together with other sequences of *S. halieti* available in GenBank. In sum, the description of the bearded vulture as a new intermediate host for *S. halieti* adds new insights on the complex epidemiology of the genus involving avian hosts.

## Introduction

*Sarcocystis* (Apicomplexa) genus comprises more than 200 species of cyst-forming obligate intracellular protozoan parasites that require a two-host prey-predator life cycle. Chronic stage as tissue cysts are developed mostly in muscular or nervous tissues of the intermediate hosts (IH); carnivore definitive hosts (DH) pray on IH and sexual phase occurs in their small intestine, giving place to oocysts/sporocysts that constitute the environmentally resistant stages and consequently serve as the source of infection for the IH (Dubey et al. 2016).

Due to the predatory and scavenger role of raptorial birds they greatly contribute to the health of ecosystems (Donázar et al. 2016); raptors are involved in the life cycle of several *Sarcocystis* species both as DH and IH (Dubey et al. 2016; Máca and González-Solís 2021). Around 30 *Sarcocystis* spp. use birds as IH, among them, at least three (*S. falcatula, S. halieti, S. wobeseri*–like) have been recognized infecting raptorial birds (Dubey et al. 2016; Prakas et al. 2021; Shadbolt et al. 2021).

While most of *Sarcocystis* infections are asymptomatic, a number of lethal cases mostly related to neurological sarcocystosis with the presence of schizonts and merozoites in such lesions have been described in raptors (Dubey et al. 2016; Maier-Sam et al. 2021); nevertheless, the global occurrence and impact of *Sarcocystis* genus in the health status of birds of prey is unknown.

Until date the only available data on the occurrence of *Sarcocystis* infections in avian species in Spain was reported by Cardells-Peris et al. (2020) who described *Sarcocystis* spp. infection by histological examination in 3 song thrushes (*Turdus philomelos*) without additional characterization in Castellón province (eastern), and by Prakas et al. (2021) who molecularly identified *S. halieti* infection in a western marsh harrier (*Circus aeruginosus*) and a black kite (*Milvus migrans*) in the northern region of Navarre.

In the present study, we identified the bearded vulture (*Gypaetus barbatus*) as a new intermediate host for *S. halieti*, a threatened species which population in the Iberian Peninsula is nowadays mainly restricted to the Pyrenees, even if numerous conservation actions have been implemented the last decades (BirdLife International, 2021).

## Materials and methods

Following the procedures included under the health surveillance program for protected species in Catalonia (NE Spain), the carcass of a black kite recovered in May 2018, and a bearded vulture found dead in December of the same year were submitted to Vallcalent Rehabilitation Center (Lleida, Catalonia). The black kite corpse (MMi18002) was found in the village of “Les Garrigues” (Lleida), with a good body condition, burns in the plantar surface of both feet, and extensive hemorrhages in the coelomic cavity, consistent with electrocution. The bearded vulture (GB18001), found in the village of “Les” (Lleida), had bilateral cataracts, dehydration, poor body condition, and a proventricular perforation caused by a bone fragment. From both animals, samples of brain, lung, heart, liver, spleen, intestine, kidney and skeletal muscle were fixed in 4% neutral buffered formalin for histopathology.

### Histopathology

Fixed tissue samples were routinely processed and 4 µm sections were stained with haematoxylin & eosin (H&E) for microscopic evaluation. In both cases, advanced autolysis harmed the evaluation of tissue sections, especially for the digestive tract, in which assessment was not possible. Nevertheless, no relevant microscopic alterations were described in the rest of the studied organ samples, except for the presence of protozoan-like tissue cysts in the skeletal muscle, which motivated the present study.

### Genetic characterization

#### DNA extraction from paraffin block-embedded tissues

Portions (10 mg in average) of paraffin block where at least 3 sarcocysts had been observed by H&E-staining were excised using sterile blades and placed in empty 1.5 mL vials. Five cycles of incubation of each sample in 1 mL of xylol at room temperature for 5 min, centrifugation at 15871 x*g* for 5 min, and the removal of the supernatant were conducted to remove the paraffin wax. This was followed by two cycles of washing in isopropanol, followed by centrifugation at 15871 x*g* for 5 min, and the removal of the supernatant. The sample was then left to dry out overnight at room temperature. DNA was extracted applying a protocol designed for skin, hair, and feathers using a lysis buffer (0.1 M Tris, 0.005 M EDTA, 0.2% SDS, 0.2 M NaCl; pH = 8.5) (Laird et al. 1991). Briefly, each tissue was incubated in 100 µL of lysis buffer with 3 µL proteinase K (Thermo Fisher Scientific, Vilnius, Lithuania) for 3 h at 56°C followed by centrifugation for 10 min at 9391 x*g*. The supernatant was transferred to a new tube and 96% EtOH was added to it. The tubes were again centrifuged for 10 min at 9391 x*g* and the 96% EtOH was replaced by 70% EtOH. After 5 min of centrifugation at 9391 x*g*, supernatant was removed, then the samples were air-dried overnight at room temperature and dissolved in 100 µL 1X TE buffer.

### PCR and sequencing

The nested PCR approach with several different primer pairs targeting ITS1 genomic region was used for the identification of *Sarcocystis* spp. (Table 1). DNA from individual sarcocysts acquired during our previous investigations (Juozaitytė-Ngugu and Prakas 2023) and nuclease-free water were used as positive and negative controls, respectively. For the amplification DreamTaq PCR Master Mix (Thermo Fisher Scientific Baltics, Vilnius, Lithuania) was used according to the manufacturer’s instructions. Cycling conditions were as: initial denaturation step for 5 min at 95°C, 35 cycles of 35 s at 94°C, 45 s of annealing at 51–65°C depending on the primer pair, 60 s at 72°C, and final extension for 5 min at 72°C. PCR products were observed in 1% agarose gel and purified using Exonuclease I and FastAP Thermosensitive Alkaline Phosphatase (Thermo Fisher Scientific Baltics). Amplified products of the second nested PCR step were sequenced directly with the 3500 Genetic Analyzer (Applied Biosystems, Foster City, CA, USA) using the same forward and reverse primers as for PCR. The obtained ITS1 sequences were truncated discarding nucleotides at the binding sites of the primers and deposited in GenBank with accession numbers OR059466–OR059469.

**Table 1:** The list of primers used for the amplification of ITS1 region of *Sarcocystis* spp

Primer name	Sequence	Nested PCR		Annealing temperature (ºC)	Product size (bp)	Target species
NITSpauk1^c^	TGTCCGGAATGGGAAGTTTT	I	External pair	51	~ 475–500	*Sarcocystis* spp.
NITSpauk2^c^	ACACCATCCDAAATTCTCAG					
NITSpauk3^c^	GGAAGGATCATTCACACGTT		Internal pair	53	~ 260–280	
NITSpauk4^c^	ATCACTGCAAGTTCCAACCA					
SU1F^a^	GATTGAGTGTTCCGGTGAATTATT	II	External pair	59	~ 1050–1150	
5.8SR2^a^	AAGGTGCCATTTGCGTTCAGAA					
GsShalF^b^	GATAATTGACTTTACGCGCCATTAC		Internal pair	65	643–647	* S. halieti*
GsShalR2^b^	CCATCCCTTTTTCTAAAGGAGGTC					
SU1F^a^	GATTGAGTGTTCCGGTGAATTATT	III	External pair	59	~ 1050–1150	
5.8SR2^a^	AAGGTGCCATTTGCGTTCAGAA					
ShalBF^c^	TTTGTGGTTGGAACTTGCAG		Internal pair	53	260–264	
ShalBR^c^	GACCTCCCCTCGACGATAAT					
ShalCF^c^	AACAACTGAATCCCCCGATA	IV	External pair	53	490–494	
ShalCR^c^	CCACTGCTAATTTCATCCCTCT					
ShalBF^c^	TTTGTGGTTGGAACTTGCAG		Internal pair	53	260–264	
ShalBR^c^	GACCTCCCCTCGACGATAAT					
SahalD1a^c^	ATGGTGTAACCAGGGGATCA	V	External pair	53	473–474	
SahalD1b^c^	CAAGACATCCATCGCTGAAA					
SahalD1c^c^	GGTCGTTCTCCTCTTTTCAGG		Internal pair	55	282	
SahalD1d^c^	TGAACAGCTTCGTTGAGACG					
SahalD2a^c^	CGGTGGCATCATCCTTTTT	VI	External pair	51	454–455	
5.8SR2^a^	AAGGTGCCATTTGCGTTCAGAA					*Sarcocystis* spp.
SahalD2b^c^	GAGGGATGAAATTAGCAGTGG		Internal pair	53	283	*S*. *halieti*
SahalD2c^c^	CAAGACATCCATCGCTGAAA					

^a^ Gjerde (2014); ^b^ Juozaitytė-Ngugu et al. (2021), ^c^ present study, NITSpauk1/NITSpauk2 and NITSpauk3/NITSpauk4 primer pairs were designed to amplify *Sarcocystis* spp. employing birds and predatory mammals as their definitive hosts

### Phylogenetic analyses

The resulted sequences of the ITS1 region were compared with those of various *Sarcocystis* spp. by Nucleotide BLAST analysis (http://blast.ncbi.nlm.nih.gov/, accessed on 20 May 2023). Different haplotypes of ITS1 of previously identified *Sarcocystis* species were determined using FaBox v. 1.5 (Villesen 2007). Multiple sequence alignments were generated using ClustalW algorithm implemented into MEGA7 (Kumar et al. 2016). Phylogenetic trees were constructed using maximum likelihood (ML) method. The bootstrap test with 1000 replicates was used to test the robustness of the suggested phylogeny.

## Results

### Histopathological findings

**Fig. 1:**
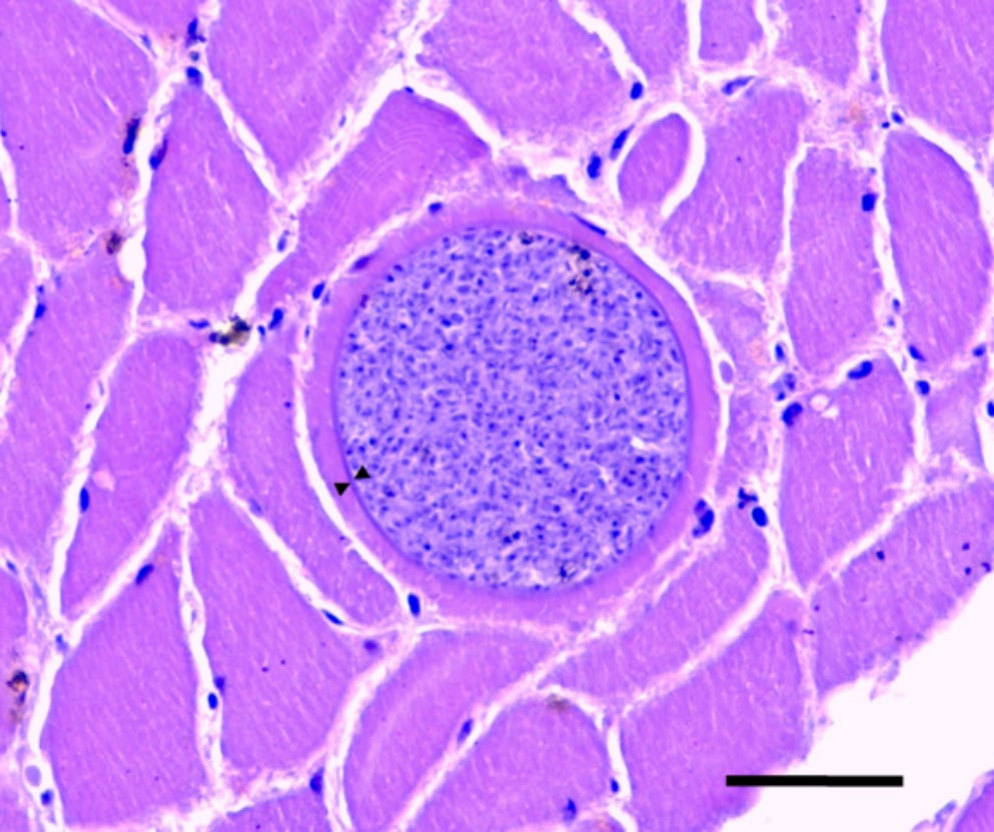
Micrograph of mature *Sarcocystis* sarcocyst in the breast muscle of a bearded vulture from Spain. Arrowheads point to the thin wall. Bar = 20 μm

*Sarcocystis*-like mature tissue cysts were observed in the tissues of both the black kite (3.4 sarcocysts/cm^2^ of tissue) and the bearded vulture (4.2 sarcocysts/cm^2^ of tissue). In both specimens, all examined cysts (n = 10) had thin (< 2 µm) wall without visible protrusions by light microscopy, apparent absence of septae and contained numerous bradyzoites (Fig. 1). No myositis nor other histological lesions were observed.

### Molecular results

Amplification was successful only using primer sets I and V (Table 1). In both cases, using external and internal primers the length of resulted fragments was no longer than 500 bp and 300 bp, respectively. The comparison of 221 bp and 241 bp sequences, obtained using NITSpauk3/NITSpauk4 and SahalD1c/SahalD1d pairs, confirmed the presence of *S. halieti* in paraffin block-embedded tissues of the bearded vulture and the black kite examined.

The 221 bp sequences obtained from both hosts were 100% identical, while 241 bp sequences differed by one SNP which was caused by A ↔ G transition. In case of 221 bp sequences, the BLAST analysis displayed 96.4–100% similarity with *S. halieti*, 95.0% similarity with *Sarcocystis* sp. (MW160469) from cinnamon skua (*Stercorarius chilensis*), 94.6% similarity with *Sarcocystis* sp. (KY348755) from Cooper’s hawk (*Accipiter cooperii*), 94.1% similarity with *Sarcocystis* sp. (MZ707151) from common raven (*Corvus corax*), 93.3% similarity with *S. corvusi* from western jackdaw (*Coloeus monedula*), and 92.4–92.8% similarity with *S. columbae* from birds of family Columbidae and Laridae. The comparison of 241 bp sequences from the bearded vulture and the black kite demonstrated 97.5–99.6% and 97.9–100% similarity with those of *S. halieti*. Higher than 90% similarity was observed when in present study generated 241 bp sequences were compared with *Sarcocystis* sp. (MZ707151) (96.3–96.7%), with *Sarcocystis* sp. (MW160469) (95.8–96.2%), with *S. columbae* (92.1–93.4%), with *Sarcocystis* sp. (KY348755) (92.6–93.0%) and with *S. corvusi* (90.2–90.6%).

**Fig. 2:**
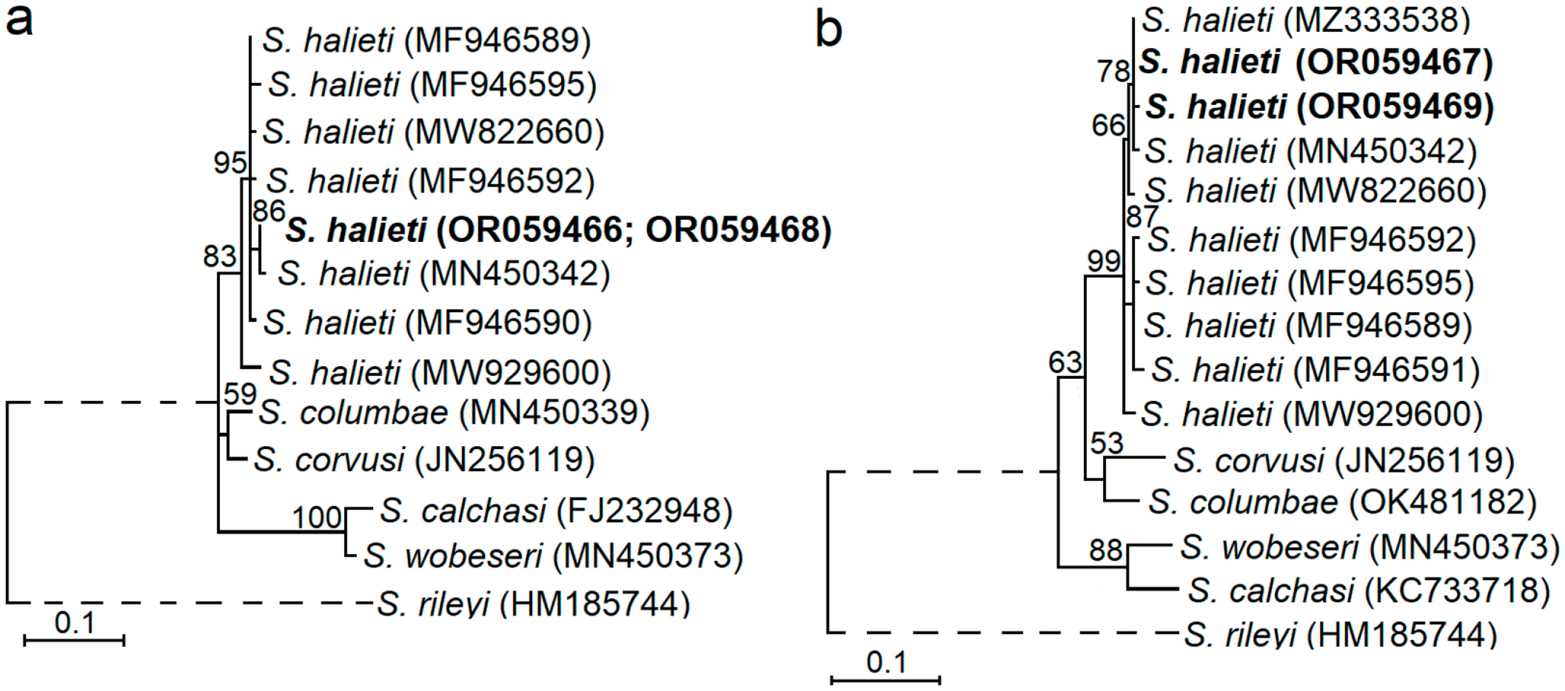
Phylogenetic analysis showing the placement of *Sarcocystis halieti* isolated from histological samples of birds of prey in Catalonia region of Spain. All possible haplotypes of *S. halieti* were included in the analysis. Trees were generated using ITS1 fragments amplified with NITSpauk3/NITSpauk4 (**a**) and SahalD1c/SahalD1d (**b**) primer pairs. Sequences were obtained from the bearded vulture (OR059466-OR059467) and the black kite (OR059468-OR059469). Based on analysis “Find Best-Fit Substitution Model (ML)” conducted in MEGA7, Tamura 3-parameter + I and Tamura 3-parameter evolutionary models were set for phylogenetic investigation

Based on the phylogenetic trees generated using ITS1 sequences and ML method, *S. halieti* isolates obtained from the bearded vulture and the black kite were placed together with other sequences of *S. halieti* available in GenBank (Fig. 2). Furthermore, despite the short-length sequences of *S. halieti* obtained, a high support (83, 99 bootstrap values) was given for the grouping of *S. halieti* isolates into one cluster.

## Discussion

Present paper constitutes the first report of *Sarcocystis* infection in bearded vulture and add new insights into the complex epidemiology of the genus involving avian hosts. A previous paper had reported the presence of *S. halieti* in the muscle tissues of black kite in Spain (Prakas et al. 2021). *Sarcocystis falcatula* and *S. calchasi* have a recognized unusual wide host rage (Dubey et al. 2016); this might be the case of *S. halieti*, given the number of recent papers reporting its occurrence in tissues of black headed gull (*Larus ridibundus*), black kite (*Milvus migrans*), common gull (*Larus canus*), common raven (*Corvus corax*), common starling (*Sturnus vulgaris*), great cormorant (*Phalacrocorax carbo*), herring gull (*Larus argentatus*), hooded crow (*Corvus cornix*), Kelp gull (*Larus dominicanus*), little owl (*Athene noctua*), Manx shearwater (*Puffinus puffinus*), Neotropic cormorant (*Phalacrocorax brasilianus*), sharp-shinned hawk (*Accipiter striatus*), western marsh harrier (*Circus aeruginosus*), and potentially the Chilean skuas (*Stercorarius chilensis*), which belong to different avian orders (Accipitriformes, Charadriiformes, Passeriformes, Procellariiformes, Strigiformes and Suliformes) (Prakas et al. 2018; Acosta et al. 2021; Juozaitytė-Ngugu et al. 2022; Llano et al. 2022; Máca and González-Solís 2022; Prakas et al. 2021; Sato et al. 2022; Juozaitytė-Ngugu and Prakas 2023). In addition, recent molecular results suggest the role of predatory birds of the Accipitridae family as DH of *S*. *halieti* (Šukytė et al. 2023).

Microscopical examination of the slides revealed the presence of thin-walled sarcocysts (Fig. 1), findings in agreement with the observations by light microscopy carried out by Prakas et al. (2020) in herring gull tissue samples. Thin-walled sarcocysts are formed by several *Sarcocystis* species characterized by a bird-bird life cycle; therefore, microscopical discrimination between these *Sarcocystis* spp. is unfeasible (Sneideris et al. 2022). In the present study we did not perform ultrastructural examination given the detailed TEM description provided by Prakas et al. (2018) for *S. halieti* in great cormorant from Lithuania. Despite the short ITS1 fragments used for molecular analysis, *S*. *halieti* was successfully identified in this study (Fig. 2). Notably, *S*. *halieti* has high intraspecific variation within ITS1, which might be related with a wide host range of this species (Juozaitytė-Ngugu and Prakas 2023; Šukytė et al. 2023). However, comparing ITS1 sequences of *S*. *halieti* with some closely related *Sarcocystis* spp. relatively low interspecific differences are observed (Juozaitytė-Ngugu and Prakas 2023). Therefore, for the within-species genetic discrimination of *S*. *halieti* more variable genetic markers should be identified.

## Conclusion

The description of the bearded vulture as a new intermediate host for *S. halieti* adds new insights on the complex epidemiology of the genus involving avian hosts and warrant the interest of further investigations to unravel the life cycles of *Sarcocystis* spp. infecting avian hosts and their impact in the health of raptorial bird populations.

## Data Availability

DNA sequences of the ITS1 marker have been deposited in GenBank with accession numbers OR059466–OR059469.
